# Ultrasound Image-Based Diagnosis of Malignant Thyroid Nodule Using Artificial Intelligence

**DOI:** 10.3390/s20071822

**Published:** 2020-03-25

**Authors:** Dat Tien Nguyen, Jin Kyu Kang, Tuyen Danh Pham, Ganbayar Batchuluun, Kang Ryoung Park

**Affiliations:** Division of Electronics and Electrical Engineering, Dongguk University, 30 Pildong-ro 1-gil, Jung-gu, Seoul 04620, Korea; nguyentiendat@dongguk.edu (D.T.N.); kangjinkyu@dgu.edu (J.K.K.); ganabata87@gmail.com (G.B.); parkgr@dgu.edu (K.R.P.)

**Keywords:** ultrasound image, malignant thyroid nodule, artificial intelligence, deep learning, weighted binary cross-entropy loss

## Abstract

Computer-aided diagnosis systems have been developed to assist doctors in diagnosing thyroid nodules to reduce errors made by traditional diagnosis methods, which are mainly based on the experiences of doctors. Therefore, the performance of such systems plays an important role in enhancing the quality of a diagnosing task. Although there have been the state-of-the art studies regarding this problem, which are based on handcrafted features, deep features, or the combination of the two, their performances are still limited. To overcome these problems, we propose an ultrasound image-based diagnosis of the malignant thyroid nodule method using artificial intelligence based on the analysis in both spatial and frequency domains. Additionally, we propose the use of weighted binary cross-entropy loss function for the training of deep convolutional neural networks to reduce the effects of unbalanced training samples of the target classes in the training data. Through our experiments with a popular open dataset, namely the thyroid digital image database (TDID), we confirm the superiority of our method compared to the state-of-the-art methods.

## 1. Introduction

Traditional disease diagnosis/treatment methods are mostly based on doctors’ expert knowledge on any given condition. However, this diagnostic method has a big limitation, that is, its performance is much more dependent on the experiences and personal knowledge of doctors. As a result, the diagnostic performance varies and is limited. With the development of digital technology, image-based diagnosis techniques have been widely used to help doctors investigate problems with organs that are underneath the skin and/or deep inside the human body [1,2,3,4,5,6,7,8,9,10,11]. For example, doctors have used X-ray imaging to capture lung and/or bone images that can help to indicate whether a disease/injury exists in these organs [9,10]. To diagnose issues with the human brain, the Computer-Tomography (CT) and/or Magnetic Resonance Imaging (MRI) techniques have been widely used [2,3]. With the help of imaging techniques, the diagnosis performance can be much more enhanced. However, the use of captured images is still dependent on personal knowledge and experiences of doctors. To overcome this problem, Computer-Aided Diagnosis systems (CAD) have been developed to assist doctors in the diagnosis and treatment processes [1,2,3,4,5,6,7,8,9,10]. As indicated by its name, the CAD systems can serve as an additional expert in the double screening process that aims to enhance the human diagnostic performance based on a computer program [11]. This kind of system uses and processes one or more captured medical images of some organs such as X-ray, CT, and MRI scans, and yields its decision that can assist doctors in diagnosing diseases. Due to its purpose, CAD systems have been widely developed and used in real-life applications such as for diagnosing the brain [2,3,7,12], breasts [4,8,13,14,15,16], lungs [10], and thyroid diseases [17,18,19,20,21,22,23,24,25,26,27,28,29,30,31,32,33,34,35].

The thyroid is an important organ located in the human neck that produces and secretes two important hormones, namely triiodothyronine and thyroxine, which are responsible for the regulation of metabolism in the human body. Due to its important role in the human body, diagnosing and treatment of thyroid disease has become important [17,18,19,20,21,22,23,24,25,26,27,28,29,30,31,32,33,34,35]. As reported in the previous studies, one important problem commonly experienced in the thyroid region is the appearance of nodules that cause thyroid cancer. Thyroid nodules are abnormal lumps that appear on the thyroid region of the human body. They could be caused by many factors, including iodine deficiency, overgrowth of normal thyroid tissue, or thyroid cancer. Thyroid nodules are usually classified into two categories based on their characteristics namely, benign cases (which are noncancerous nodules), and malign cases (which can cause thyroid cancer) [36]. In both the benign and malign cases, the appearance of thyroid nodules can cause problems with patient health. With the appearance of nodules, the thyroid region can be malfunctioned. Although the benign case has little effects on patient health, it can cause aesthetic problems and/or make it difficult for the patients to breathe and/or swallow. The malign case can cause thyroid cancer. Fortunately, most detected thyroid nodules are benign cases as reported in the previous studies [19,36]. However, diagnosing and treating malign cases is still very important.

There have been several methods of diagnosing thyroid nodules such as physical examination, thyroid function tests, and Fine Needle Aspiration (FNA) biopsy. The physical exam is normally done at the first stage of the diagnosis process in which the patients are asked to perform several physical tests on the thyroid region such as swallowing to check the shape, size, and the movement of nodules. However, this method is just a primary test and normally does not give deep information about the nodules’ condition. To gain a deep look inside the thyroid problem, thyroid function tests or FNA are normally invoked. In the thyroid function test method, the level (amount) of the two hormones (thyroxine and triiodothyronine, which are produced by the thyroid region) is measured to see whether there is any abnormality in thyroid functionality. FNA can also be applied in diagnosing thyroid nodules to produce good diagnosis results. However, these methods required are labor-intensive, invasive, and costly. As an alternative, image-based thyroid nodule diagnosis has been used in various applications. This method uses high-frequency sound waves (ultrasound wave) to produce images of the thyroid region. As a result, this method provides rich information of thyroid nodules such as the shape and structure of nodule as well as the condition of the nodules.

Using the ultrasound thyroid nodule images, there have been several previous studies on CAD for the thyroid nodule detection and classification problems. In contrast to the conventional thyroid diagnosis methods mentioned above, the CAD methods for thyroid nodules use ultrasound thyroid nodule images as inputs and produce thyroid nodule regions and/or the status of nodules (benign or malign) [17,18,19,20,21,22,23,24,25,26,27,28,29,30,31,32,33,34,35,36]. Similar to normal image processing systems, CAD systems for thyroid nodule use several image processing techniques to extract information from input images for detection/classification purposes. Based on the methods for extracting information from images, the previous studies can be categorized into three groups: the group using handcrafted feature extraction methods, the group using deep feature extraction methods, and the group that is a fusion of the two.

Handcrafted-based image feature extraction methods have been widely applied for a long time, especially with the simple image-based systems and/or before the appearance of deep learning-based techniques. As indicated by its name, this kind of method uses several handcrafted image feature extraction methods that are designed by experts based on their knowledge of specific problems to extract efficient features from input images for image-based processing systems. For the thyroid nodule CAD, the handcrafted feature-based method has also been used previously [22,24,31]. Chang et al. [24] used up to 78 texture features extracted from ultrasound thyroid nodule images for the thyroid nodule classification problem. Based on the extracted image features, they used Support Vector Machines (SVMs) to classify input images into several categories such as nodule versus non-nodule and follicles versus fibrosis. Sudarshan et al. [22] used wavelet transform to analyze the input ultrasound thyroid images for the thyroid nodule classification problem. A similar approach, Raghavendra et al. [31] used the segmentation-based fractal texture analysis technique to analyze ultrasound thyroid images under different threshold values for the classification problem. Ouyang et al. [26] found that linear and non-linear classifiers yield similar classification results for the thyroid nodule classification based on handcrafted image features. Since the handcrafted image feature extractors were designed and selected by expert knowledge of authors, they only reflect some limited aspects of the problem. As a result, the classification performance is limited.

With the development of technology, such as the back propagation algorithm, neural network, and Graphics Processing Units (GPUs), the deep learning-based technique has recently been applied to solve many problems in medical image processing systems [1,2,10,12,21,36]. For the thyroid nodule detection/classification problem, the deep learning-based method has gained a lot of success. As indicated by its name, the deep learning-based method, such as Convolutional Neural Network (CNN), automatically learns the useful texture features for the detection/classification problem instead of using handcrafted (fixed) feature extraction methods. As a result, the deep learning-based method can produce more superior results than handcrafted-based methods. In a study by Zhu et al. [21], they proposed a method for thyroid nodule classification using CNN systems. In their study, they fine-tuned the residual network (ResNet18-based network) and obtained good classification results using a public dataset. Similar to the work by Zhu et al., the work by Chi et al. [23] also used the CNN network to classify ultrasound thyroid nodule images into benign and malign nodules. However, different from the study by Zhu et al. [21], Chi et al. [23] used the GoogLeNet for a classification purpose. In addition, they trained their CNN model using two datasets to reduce the effect of the over-fitting problem and the variation of input images. In a study by Sundar et al. [28], the authors proposed a general framework for thyroid nodule classification using the CNN network, including the fine-tuning, training from scratch, and the use of pretrained networks for image feature extraction. With their proposed methods, they performed various experiments using two popular CNN architectures, including a relative shallow network based on VGG16-Net architecture, and a deep network based on Inception (GoogLeNet) architecture. In some other studies, the thyroid nodule classification can also be done by a detection-and-classification approach as shown in a study by Song et al. [27]. In that study, Song et al. used a detection network such as multiscale single-shot detection network (multiscale SSD) or Yolo network to roughly detect the position of thyroid nodules. Additionally, then, they performed the nodule classification using the detection results of the first step. This method has the advantage that noise and non-nodule regions can be removed before performing the classification step. However, it is difficult to find small nodules, and the network architecture is very complex.

As a fusion of the two mentioned approaches, there exist studies that combine the handcrafted and deep learning methods to enhance the classification performance. In a study by Nguyen et al. [36], they found that the information in the frequency domain can be useful for discriminating easy samples of benign and malign cases, and the deep learning-based method can be useful for discriminating harder samples (ambiguous samples). Based on this observation, they proposed a method that applies a cascade classifier scheme for the thyroid nodule classification problem. As a result of their study, they showed that the combination of handcrafted and deep features is efficient for enhancing classification accuracy compared to the use of individual feature extraction method. The following are more detailed differences between previous study [36] and our research. First, one CNN of ResNet was used in a previous study [36], but multiple CNNs of ResNet and InceptionNet are used in our research. Second, only the binary cross-entropy loss was used in a previous study [36] whereas only the weighted binary cross-entropy loss was newly adopted in our research. The weighted binary cross-entropy loss function is efficient for reducing the overfitting problem caused by the unbalanced training samples of the target classes in the training data. Third, the final classification of thyroid nodule was performed based on the one output score of ResNet in a previous study [36]. However, the outputs of multiple CNNs of ResNet and InceptionNet are combined by score level fusion in our research.

There is a common limitation in the aforementioned studies: they did not fully consider the problems associated with deep learning-based methods, such as the imbalance of training image samples, the depth of the network, and the variation of the size of objects. For example, the classification model can produce biased results if the training data have an imbalance of samples in target classes, or it is difficult to construct a very deep network that can capture features of both the small and large sizes of objects. In Table 1, we summarized the previous studies for the thyroid nodule classification problem in comparison with our proposed method. To overcome this limitation, we propose a novel approach for the thyroid nodule classification problem by modifying the loss function of a conventional CNN network and a combination of multiple CNN networks to enhance the learning ability of the deep learning method. In comparison with the methods in the previous studies, our proposed method is novel in the following four ways:

- We propose the use of multiple CNN-based models to analyze input ultrasound thyroid images deeply for the classification problem. Since each CNN model has its own architecture and characteristics of learning the characteristics of input images, the use of multiple CNN-based models can help to extract richer information compared to using an individual model.
- In order to solve the problem of unbalanced data samples between the benign and malign classes in the training data, we propose the use of a weighted binary cross-entropy loss function instead of the conventional binary cross-entropy loss function. As the name suggests, we assign a higher weight value to data samples of class (benign or malign), which have a smaller number of data than the other. This procedure helps increase the focus of the training process on this class rather than the other class. As a result, it helps to reduce the effects of the overfitting problem of the CNN networks when training with unbalanced data.
- We combine the outputs of multiple CNN-based models to enhance the classification performance using several bagging methods, including MIN, MAX, and AVERAGE combination rules which take the minimum, maximum, and average results of the multiple CNN-based models, respectively.
- We make our algorithm available to the public through [37], so that other researchers can make fair comparisons.

The remainder of our paper is organized as follows. In Section 2, we provide detailed descriptions of our proposed method for diagnosis of malignant thyroid nodules using the artificial intelligence technique. In Section 3, we validate the performance of the proposed method using a public ultrasound thyroid dataset, namely the Thyroid Digital Image Database (TDID) dataset [20]; compare the findings with the previous studies; and provide a discussion about our results. Finally, we present the conclusion of our study in Section 4.

## 2. Proposed Method

### 2.1. Overview of the Proposed Method

**Figure 1:**
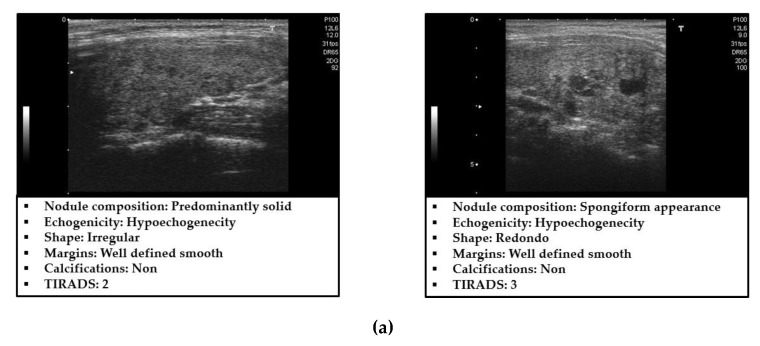

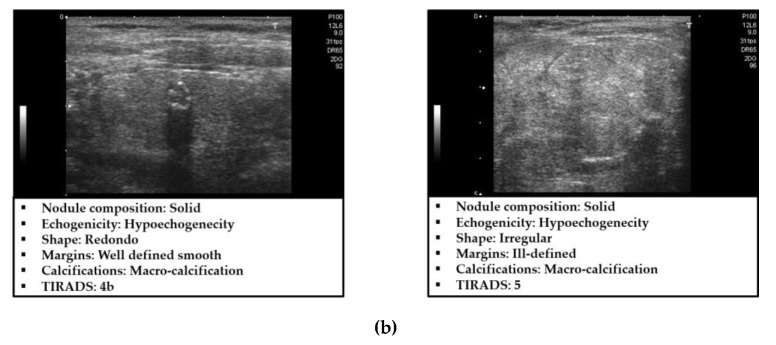
Example of captured ultrasound thyroid images in the thyroid digital image database (TDID) dataset [20]: (**a**) benign cases and (**b**) malign cases.

**Figure 2:**
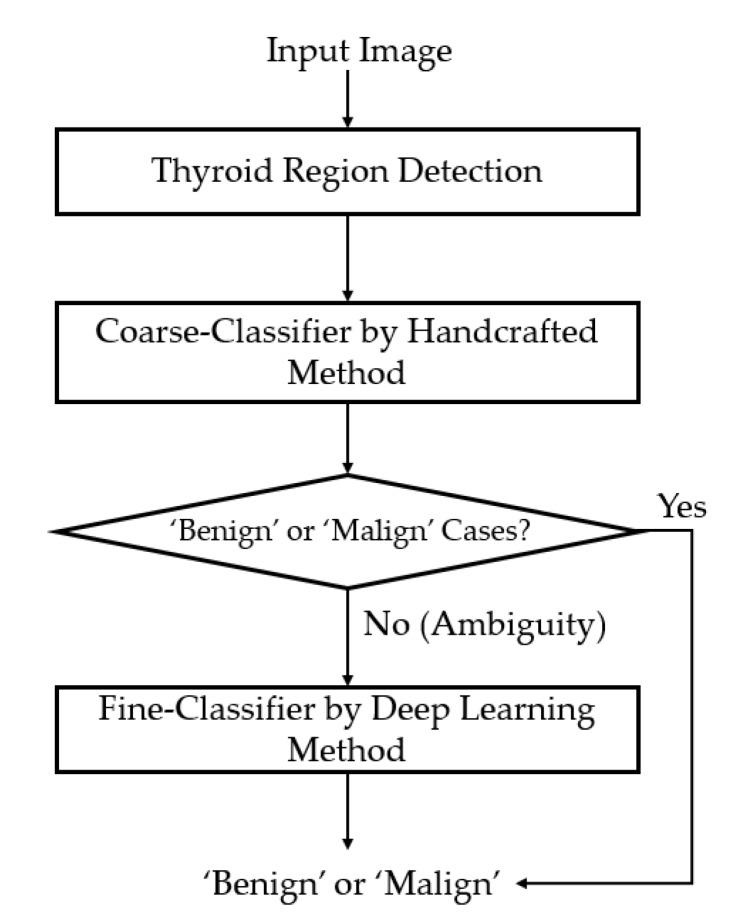
Flow chart of the proposed method.

In Figure 1, we show some examples of ultrasound thyroid images in the TDID dataset [20]. As shown in these examples, the captured ultrasound thyroid images contained two main regions: the background (dark region) and the thyroid (brighter regions). Focusing on the thyroid region, the benign and malign cases exhibited several differences: the malign case images contained nodules with round-like shape and exhibited the calcification phenomenon. Based on this observation, we proposed a new thyroid nodule classification method as shown in Figure 2.

In Figure 2, we depicted the overall flow-chart of our proposed method for thyroid nodule classification using ultrasound images. As shown in this figure, our proposed method receives an ultrasound image of thyroid region and outputs a suggestion for doctors (radiologists) about whether the image contains a benign or malign case of a thyroid nodule. To perform its functionality, an input ultrasound thyroid image is first passed through a thyroid region detection method to filter-out the background and noise region before feeding it to our main algorithm. This step is necessary and important to enhance the classification performance because the background and noise provide redundant information, and consequently, they can have negative effects on the classification system. The detail description of this step is mentioned in Section 2.2. As a subsequent step, we performed a coarse classification step to classify the input ultrasound thyroid image into one of three categories, including ‘benign’, ‘malign’, and ‘ambiguous benign–malign’, using the image of the thyroid region (image obtained after filtering out the background and noise) based on a handcrafted-based method extracted in the frequency domain. This classification step was used to detect the easy benign or malign samples, reduce processing time, and shift the focus of the deep learning-based model on the more difficult samples. When the coarse classifier classified the input images as ‘ambiguous benign–malign’ cases, the input image was then further processed (classified) by a deep neural network based on the CNN method. The detail descriptions of these steps are included in the Section 2.3 for the coarse classifier, and Section 2.4 for the fine classifier.

### 2.2. Preprocessing of Captured Thyroid Images

**Figure 3:**
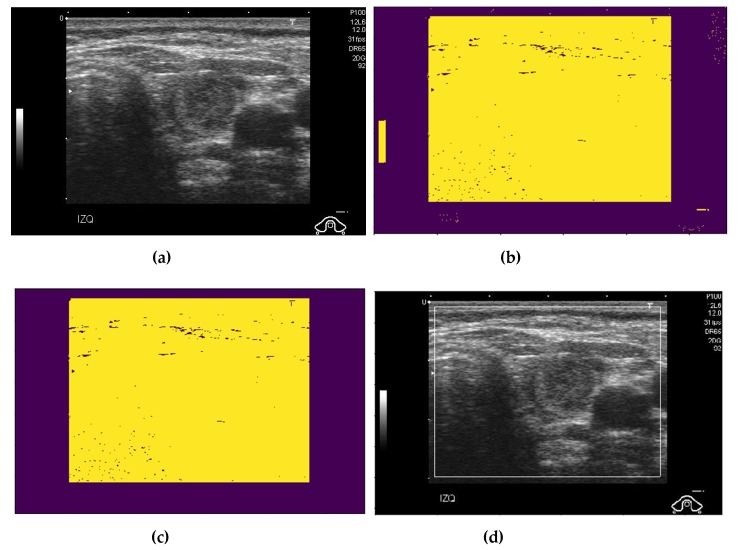
Example result of the thyroid region detection algorithm used in our study: (**a**) an input ultrasound thyroid image; (**b**) the binarized image; (**c**) the thyroid region detection by selecting the largest object; and (**d**) the final detection results.

As shown in Figure 1 and Figure 2, the captured ultrasound thyroid images contained two main parts, that is, the background (boundary parts with low illumination and some additional artifacts) and the thyroid region (the inner brighter part that captures the details of the thyroid region). It is easy to see that the background regions contain no information about whether an image contains benign or malign cases of thyroid nodules. Besides, it also contains some artifact information that was added to an image as indicators for the radiologist, such as the patient information or capturing system configuration, during the image acquisition process. Due to this reason, the background region should be removed before passing images to the main classification system. This step is a preprocessing step and has been well-studied in a previous study [36]. In our study, we used a popular algorithm for removing background regions as shown in the studies by Zhu et al. [21] and Nguyen et al. [36]. Steps for localizing the thyroid region and removing the background regions are roughly described in Figure 3.

As shown in Figure 1, the thyroid region is normally displayed as the largest brighter region in the captured ultrasound thyroid image. Although several brighter regions exist in an ultrasound thyroid image, such as the illumination indicator and text for specifying capturing system configuration, the size of these regions is much smaller than that of the thyroid region. Based on this observation, we first performed an image binarization method to detect all brighter regions in the captured image using an optimal threshold value. In our study, we used a binarization method proposed by Otsu’s et al. [38], which takes an input image and performs binarization adaptively by selecting the most suitable threshold value. A result of this binarization step is given in Figure 3b using the input image of Figure 3a. As shown in Figure 3b, although there were some brighter regions detected, the thyroid region had the largest size. Based on this truth, we detected the thyroid region by selecting the largest object in the binarized image and discarding the other regions as shown in Figure 3c. Finally, the detected thyroid region was determined by taking the bounding-box in the input image (in Figure 3a) based on the selected region of Figure 3c. An example of a resultant image of this step is given in Figure 3d using the input image of Figure 3a. As we can see from this example, the thyroid region was well localized using our localization method.

### 2.3. Coarse Classifier Based on Information Extracted in the Frequency Domain

**Figure 4:**
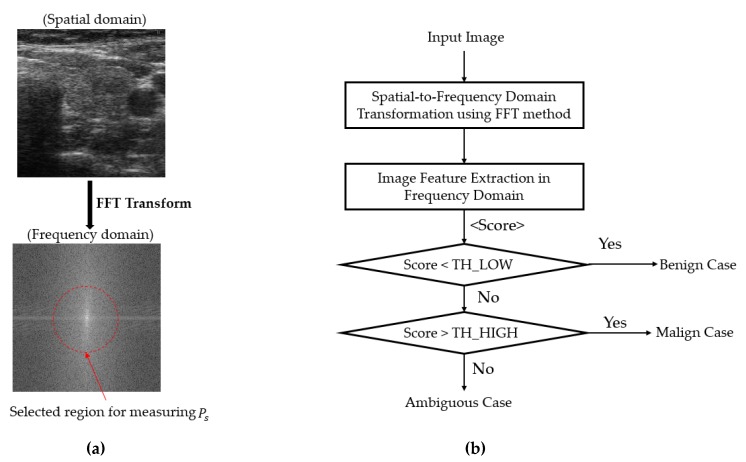
Coarse classifier based on information extracted in the frequency domain using the Fast Fourier Transform (FFT)-based method: (**a**) a thyroid image represented in spatial and frequency domain with a selected circle frequency regions and (**b**) the flowchart for classifying thyroid images into ‘benign’, ‘malign’, or ‘ambiguous’ region in our study.

As shown in Figure 2, our proposed method is based on a cascade structure of classifiers using handcrafted-based and CNN-based methods. As the first stage of our proposed classification method, we performed a coarse classification based on the information extracted from images in the frequency domain, as suggested by Nguyen et al. [36]. The purpose of this classifier is to preclassify samples that are easily classified using information extracted in the frequency domain. As exploited by Nguyen et al. [36], there are differences between benign and malign case images in the frequency domain caused by the appearance of nodules and calcification phenomenon in the thyroid regions. That is, the appearance of nodules and calcification makes the captured ultrasound thyroid image of malign cases brighter, and the change in pixel values is faster around these nodule regions than other regions. Based on this observation, we used the Fast Fourier Transform (FFT) method to extract this difference and classify an ultrasound thyroid image into one of three categories: ‘benign’, ‘malign’, and ‘ambiguous benign–malign,’ as shown in Figure 4. As shown in Figure 4a, the thyroid region image was first transformed from the spatial domain to the frequency domain using the FFT method to extract the distribution of image energy in the frequency domain. With this extracted image in the frequency domain, Nguyen et al. proposed an image feature extraction method that uses the ratio between some selected frequency components and the total frequency components as shown in Equation (1) [36]. In this equation, *P_s_* indicates the total power spectrum of image frequency components inside a selected frequency region, and the *P* indicates the total power spectrum of all frequency components of an image [36]. As indicated by Nguyen et al. [36], there could be several methods for selecting the frequency region in which we used to measure *P_s_* (the selected frequency components) such as the use of frequency components inside a circle, horizontal, vertical, or a combination of them [36] around/through the DC component (zero-frequency component) of an image. However, as indicated by their work with the TDID dataset, they showed that the frequency components inside a circle around the DC component works better than other methods. Therefore, we selected to use the circle shape in our study as shown in Figure 4a (red circle).

(1) $$\mathrm{Score}=\frac{P_{s}}{P}$$

We compared the extracted image feature in the frequency domain with two threshold values, i.e., TH_LOW and TH_HIGH in Figure 4b for classifying the input ultrasound thyroid image into one of the three categories. These threshold values are experimentally obtained based on the training dataset. As a result, if the extracted image feature (Score in Figure 4b) is lower than TH_LOW, it is regarded as the ‘benign’ case image; if the extracted image feature is higher than the TH_HIGH image, it is regarded as the ‘malign’ case image. Otherwise, it is considered to belong to the ‘ambiguous benign–malign’ category in which we are not sure which class it should belong to. For this case, the final classification was done based on our second classifier that was based on the deep learning technique.

### 2.4. Fine Classifier Based on a Combination of Multiple CNN Models

#### 2.4.1. Introduction to the Deep Learning Framework

**Figure 5:**
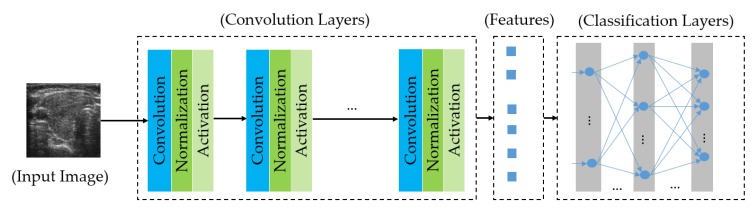
A general architecture of a Convolutional Neural Network (CNN) network for the image classification problem.

The deep learning-based method implies the use of a deep (many layers) neural network for a regression or classification problem. Although this is not a new technique, this method has recently attracted lots of attention from researchers because of the development of GPUs that are used to speed-up the processing of the network, and lots of superior (state-of-the-art) performances of digital signal processing systems have been reported [39,40,41,42,43,44,45,46,47,48,49,50]. This kind of signal processing technique has been successfully and widely used in many fields including image processing [39,40,41,42,43,44,45,46,47,48] and natural language processing [49,50]. In Figure 5, we show the general architecture of a CNN network, which is a special kind of deep learning-based technique and has been successfully used for the image classification problem. As shown in this figure, a CNN network is composed of two main components, including a feature extraction component based on convolution operation, and a classification component based on a multilayer perceptron (neural) network. This structure allows us to learn efficient representation (image texture features) of an input image using the filtering technique through the application of convolution operation. With the extracted image features, it is possible to learn a classifier to classify input image into predesigned classes. All of the network parameters (weights and biases of convolution filters and multilayer perceptron) can be trained and automatically obtained by a training process using a back propagation technique and training data. This is the key to make the learning-based method outperform the handcrafted-based method for the image-based classification problem. In addition, the use of convolution operation with a weight-sharing scheme allows us to construct a deeper network than the conventional neural network.

According to the type of applications, a suitable CNN network was used. Lots of CNN architectures have been proposed for various image-based systems such as image classification [39,40,41,42,43], object detection [44,45], 3D image reconstruction [46], and image feature extraction [47,48]. Although the CNN network has been successfully used in many image-based processing systems, it still has several limitations caused by its characteristics and internal structure. As mentioned in the previous studies [39,40,41,42,43], there are two main problems frequently associated with a CNN network. The first problem is caused by the depth of the network. To learn from data efficiently, we normally need to construct a deep network that contains many weight layers. However, the deep network is normally difficult to train due to the vanishing gradient problem [41]. The second problem is caused by the huge number of parameters that need to be learnt through the training process. For an image classification system, many CNN networks have been used, such as AlexNet, VGGNet, ResNet, DenseNet, and InceptionNet. According to their structures, the AlexNet contained about 62 million parameters, the VGGNet-16 contained about 138 million parameters, the VGGNet-19 contained about 143 million parameters, etc. To learn these huge amounts of parameters requires a strong hardware power (Central Processing Unit (CPU), GPU) as well as a large amount of training data. These problems can have strong negative effects on the performance of medical image-based systems because we normally require high performance systems using less training data. This is because it is difficult to collect a large number of medical images owing to special characteristics of this kind of images: they require expensive data acquisition devices and the cooperation of patients.

**Figure 6:**
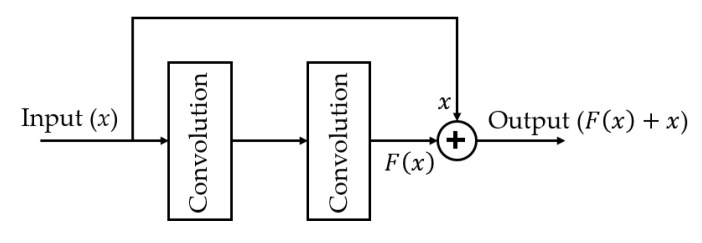
Methodology for constructing the residual convolution block.

As explained above, the conventional CNN networks such as AlexNet or VGGNet were constructed by chaining weight layers (convolution and dense layers) to extract image features and learn classifiers for the classification problem. This is a basic CNN architecture and it works fine for a not-too-deep network. However, there is a problem, called vanishing gradients, which can occur when the depth of the network increases, and this problem makes the network difficult to train and consequently degrades the classification performance [41]. To solve this problem, He et al. [41] propose a new method for not only constructing a very deep CNN network, but also making it easier to train, namely the residual network (ResNet). In Figure 6, we described the methodology of the ResNet network building block. By using a new kind of connection, called skip connection, this new type of CNN architecture can make the network skip some training layers when the input and output of these layers are close to the identification function. As a result, the network is deeper and easier to train compared to the conventional CNN networks. In our study, we used this type of CNN architecture to construct a very deep network for learning texture feature of input ultrasound thyroid images. In detail, we used a ResNet50-based network that contained a total of 50 weight layers for our classification problem as explained in Section 2.1 and Figure 2.

**Figure 7:**
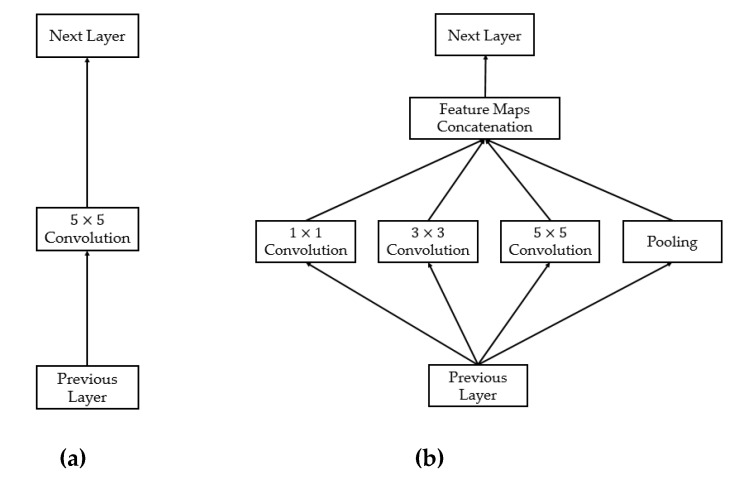
Comparison between: (**a**) the conventional convolution block versus (**b**) the naïve inception block.

Although the conventional CNN networks have successfully been used to capture image texture features, their performances are still affected by the large variation of the size of objects that appears in input images. As a result, choosing a right kernel size for the convolution mask is difficult to achieve, and normally a single optimal size of convolution mask does not exist. To solve this problem, Szegedy et al. [42] proposed a new network structure that applies multiple sizes of the convolution mask to extract image features from the input image. This new network structure is done by stacking its building blocks, namely inception blocks, as shown in Figure 7. As shown in this figure, instead of using a single convolution operation between a previous layer and the next layer as has been used in conventional CNN (Figure 7a), the inception block performed various convolution operations with various kernel sizes as shown in Figure 7b. Figure 7b shows the naïve inception block to demonstrate the methodology of the inception method in which the output feature maps are obtained by concatenating the outputs of several convolution and pooling layers [42]. Obviously, we could extract texture information at various object sizes (scales) by using multiple convolution operations at different sizes of the convolution kernel. As we could observe from this figure, the feature map at the output of the inception block was much richer in information than the conventional convolution block. This structure is not our contribution, but was proposed by the authors of the inception network [42].

#### 2.4.2. Proposed Method for Thyroid Nodule Classification Using Multiple CNN Models

**Figure 8:**
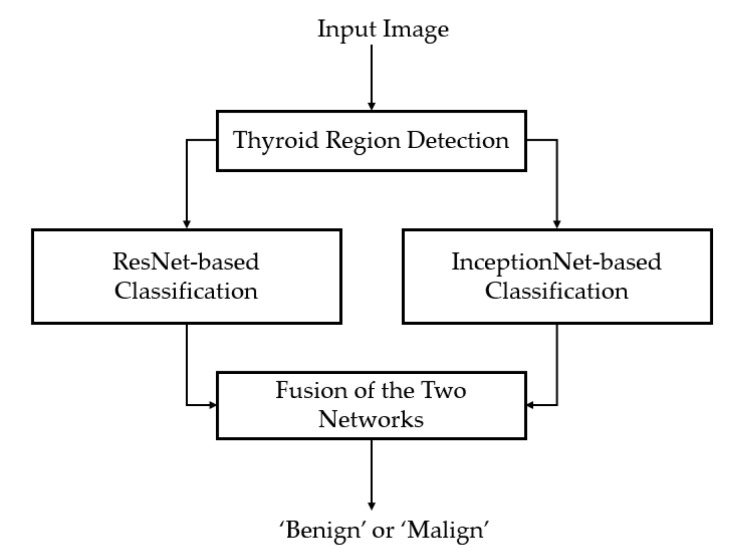
Flow-chart of the deep learning-based system constructed by combining classification results of multiple CNN networks.

As the second classifier used in our proposed method, the deep learning-based method was applied in the case of the first classifier producing an ‘ambiguous benign–malign’ case as its answer. This result indicates that the input thyroid ultrasound images were difficult to classify based on the first classifier and needs to be processed further by the second classifier. In our study, we proposed the use of a combination of multiple CNN models for the classification purpose as shown in Figure 8.

As shown in this figure, we tended to enhance the classification performance of single CNN model by combining the classification results of multiple models that have different network architectures. For this purpose, we used two efficient CNN architectures, including the residual network and inception network, as explained in Section 2.4.1 and Figure 6 and Figure 7. As shown in the previous studies, the residual network works well for thyroid nodule classification [21,36]. Therefore, we used this network in our study. Besides, in our study for the thyroid nodule classification problem, the nodule’s size was varied according to the condition (status) of the thyroid nodules. To reduce the effect of this variation on our classification algorithm, we also used the inception network to learn the characteristics of thyroid nodules. In Table 2 and Table 3, we show the detailed descriptions of the ResNet50-based and Inception-based network architectures used in our study. By combining the results of these two networks, we could enhance the classification result for CAD for the thyroid nodule classification system.

To combine the results produced by ResNet50-based and Inception-based network, we used three combination methods, including the MIN, MAX, and SUM rules as shown in Equations (2)–(4). These combination methods have been widely used to combine classification scores of multiple biometric models or multiple classifiers for a single biometric [51,52,53]. In detail, at the output of each network we could obtain a classification score that presents the probability of the input image belonging to the benign or malign class. We referred to S_1_ and S_2_ as the decision scores produced by the ResNet50-based model and the Inception-based model, respectively. Then the MIN rule was performed by selecting the smallest score between these two scores; the MAX rule was performed by selecting the largest score between the two scores; and the SUM rule was performed by taking the average score of the two scores. If the final combination score was larger than the predetermined threshold, the input image was classified as the benign class. If not, it was regarded as the malign class. This optimal threshold was experimentally determined with training data.

(2) $$\mathrm{MIN}=\min\left(S_{1},S_{2}\right)$$

(3) $$\mathrm{MAX}=\max\left(S_{1},S_{2}\right)$$

(4) $$\mathrm{SUM}=\frac{S_{1}+S_{2}}{2}$$

#### 2.4.3. Weighted Binary Cross-Entropy Loss Function for Compensating the Imbalance of Training Samples

For the image classification problem, the previous studies mostly used the cross-entropy function to measure the loss (the difference between ground-truth and predicted labels) function [39,40,41,42]. In our specific case of thyroid nodule classification, the cross-entropy function is reduced to binary cross-entropy because we only have two classes of benign and malign. As a result, the formula for the loss function using Binary Cross-Entropy (BCE) is shown as Equation (5). In this equation, *y* and *(1-y)* indicate the ground-truth labels of two classes (benign and malign); and *p* and *(1-p)* indicate the predict labels (probability) of these classes. This is a very nice loss function that incorporates the probability theory into its calculation. However, this function only works well if the number of training data of the two classes is balanced because it considers the losses caused by each class equally. In the case of imbalance in the training data of the two classes such as the medical image processing system that is normally faced with the problem of data collection due to special characteristics of medical images, the binary cross-entropy function can produce bias in the trained classifier. To solve this problem, our proposed method uses a modified version of the binary cross-entropy, called the Weighted Binary Cross-Entropy (wBCE), as shown in Equation (6). As shown in this equation, we assigned different weight values to the losses caused by samples in each class in the binary cross-entropy function. As a result, the weighted cross-entropy function makes the training process to focus more on the class, which has a small number of samples than the other classes, and consequently reduces the bias in the trained classifier. The weight values can be determined experimentally based on the actual number of samples in each class in the training dataset. In our experiments with the TDID dataset, the optimal weights of $w_{0}\;$(0.7) and $w_{1}$ (0.3) were determined with the training data, and this result corresponds to the fact that the number of malign samples is much larger than the number of benign samples. In addition, we show that the weighted binary cross-entropy function is sufficient to reduce bias in the classification results in Section 3.3.

(5) BCE=−ylog(p)−(1−y)log(1−p)

(6) $$\mathrm{wBCE}=-w_{0}\mathrm{ylog}\left(p\right)-w_{1}\left(1-y\right)\log\left(1-p\right)$$

The weighted cross-entropy loss function is not a new method to deal with the imbalanced data problem in a deep learning-based classification system. There exist several similar studies focusing on the problem such as the use of focused anchors loss [54], focal loss [55], and class-balanced loss [56]. In these studies, the main idea is that they can down-weight the well-classified examples to make the classification networks focus on the hard sample ones (focal loss, focused anchors loss); or assign weights to samples according to the volume of classes (class-balanced loss). As stated in our paper, the medical image processing systems normally face a common problem caused by the lack of training data. Due to this problem, the imbalanced data problem is normally occurred and consequently reduces the performance of the classification system. Therefore, the use of weighted cross-entropy loss function in our study can be seen as a simple application of this type of technique that is applicable to enhance the performance of the medical image processing systems.

## 3. Experimental Results

Based on the proposed method explained in Section 2, in this section, we present various experiments with a public ultrasound Thyroid Nodule Image Dataset (TDID dataset) to measure the classification performance of our proposed method. The experimental results are given in the subsections as follows.

### 3.1. Dataset and Experimental Setups

Although studies for the ultrasound image-based thyroid nodule classification problem exist [21,22,23,24,25,29], most of the datasets used in these studies are private. In addition, it is very difficult to collect a large amount of data owing to the lack of time and the special characteristics of the medical problems, in which expensive image collection systems and the patient’s cooperation are required. Therefore, we decided to use a public thyroid nodule image dataset, namely the Thyroid Digital Image Database (TDID), which was collected and published by Pedraza et al. [20] at the Universidad Nacional de Colombia. This dataset has been widely used in the previous studies for the thyroid nodule classification problem [21,28,36]. Therefore, we can not only evaluate the classification performance of our proposed method, but also compare it with lots of the previous studies to investigate the efficiency of our study.

The TDID dataset was published in 2015 and contains ultrasound thyroid images of 298 patients. For each patient, one or more ultrasound images of the thyroid region were collected in the RGB format with the image size of 560 pixels × 360 pixels. As a result, we extracted a total of 450 thyroid nodule images for our experiments. To assess the condition of the thyroid region, a Thyroid Imaging Reporting And Data System (TI-RADS) score is given for each image that was evaluated by radiologists. The TI-RADS score is defined as a standard to evaluate the condition of thyroid nodules and can take one among seven possible values of {1, 2, 3, 4a, 4b, 4c, and 5}. Among these possible values, the TI-RADS score of 1, 2, and 3 indicate that the thyroid nodules are normal (TI-RADS score of 1), benign (TI-RADS score of 2), and no suspicious ultrasound features (TI-RADS score of 3), respectively. As indicated by their meaning, ultrasound thyroid images with TI-RADS scores of 1, 2, and 3 were grouped together to indicate that they belong to the benign case. The other four possible values of 4a, 4b, 4c, and 5 indicate that the thyroid nodule has one, two, three, and five suspicious features, respectively. Due to their meaning, the thyroid nodule images with these four TI-RADS scores were normally grouped together to indicate the malign case of thyroid nodule. In our study, we also used the TI-RADS score to preclassify thyroid images into either the benign or the malign category for the classification problem (ground-truth labels).

As explained in Section 2, our proposed method was based on a learning framework to determine the best classifier for the classification problem. Therefore, we divided the TDID dataset into the training and testing dataset for this purpose. In detail, we used a five-fold cross-validation scheme to train and measure the performance of our classification system. As a result, we randomly divided the benign and malign case data into five parts. Among these five parts, four parts were assigned as the training data, and the remaining part was assigned as the test data in the 1st fold validation. This process was repeated five times to train and measure the performance of our proposed method as a five-fold cross validation scheme. Then, the average testing accuracy of five folds was determined as a final testing accuracy. In Table 4, we show the detail information of our experimental data in TDID dataset. Although a validation set is usually used during the training process of a neural network, we did not use a validation set in our experiments. The reason is that the number of images in the TDID dataset was small consisting of 450 images. Even we could split this dataset into training/validation/testing sets, this division method consequently reduced the size of training and testing sets, which could result in the insufficiency of training a neural network, and we used only training and testing sets in our experiments like previous methods [21,28,36]. To train the CNN models mentioned in Section 2.4.2, we performed the fine-tuning technique to reduce the effects of under- or overfitting problem. The parameters for the training process are given in Table 5.

### 3.2. Criteria for Classification Performance of a Thyroid Nodule Classification Method

To measure the performance of a thyroid classification system, there are three popular metrics that have been in use, including the sensitivity, specificity, and the overall classification accuracy [21,23,28,36,57]. Similar to the previous studies, we also used these three performance measurements in our experiments to measure the performance of our proposed method as well as to compare our classification performance with the previous studies. Formulas for these measurements are given in Equations (7)–(9). Since CAD for a thyroid nodule classification system normally focuses on two different aspects of the classification problem, that is, the correct classification of benign case images and a correct classification of malign case images, the specificity and sensitivity measurements were used to measure the accuracy of these aspects. First, the sensitivity was measured as the ratio between the true positive (*TP*) samples (samples that are malign case images are correctly classified as the malign ones) over the total number of the malign cases image (*TP* + false negative (*FN*)) in a test dataset as shown in Equation (7). Second, the specificity is the measurement of true negative (*TN*) samples (samples that are benign cases are correctly classified as benign ones) over the total number of benign case images (*TN* + false positive (*FP*)) in a test dataset, as shown in Equation (8). As their definition and measurement methods, the sensitivity reflects the ability of a classification system in correctly detecting malign cases, while the specificity reflects the ability of a classification system to correctly detect (classify) benign cases. To access an overall (average) ability of the classification system, the third measurement (overall accuracy) was used and measured by the total number of correct classification/detection samples (true positive and true negative samples) over the total number of samples in a test dataset as shown in Equation (9). As indicated by the above explanations, high values of specificity, sensitivity, and accuracy were expected for a good classification system. In our experiments, we measured these criteria by using our proposed method with the TDID public dataset for performance measurement and comparison with other studies.

(7) $$\mathrm{Sensitivity}=\frac{\mathrm{TP}}{\mathrm{TP}+\;\mathrm{FN}}$$

(8) $$\mathrm{Specificity}=\frac{\mathrm{TN}}{\mathrm{TN}+\;\mathrm{FP}}$$

(9) $$\mathrm{Accuracy}=\frac{\mathrm{TP}+\mathrm{TN}}{\mathrm{TP}+\mathrm{TN}+\mathrm{FP}+\mathrm{FN}}$$

### 3.3. Classification Results Based on Multiple Artificial-Intelligence Models

As our first experiment, we measured the classification performance of the proposed deep learning-based network for the thyroid nodule classification problem. For this purpose, we first performed experiments using individual CNN network, i.e., ResNet50-based architecture as shown in Table 2, and Inception-based architecture as shown in Table 3. As shown in Section 2.4.3, we set the weight values of weighted binary cross-entropy w_0_ and w_1_ as 0.70 and 0.30, respectively, because the number of training samples in the benign cases is much smaller than the number of training samples in the malign case in the TDID dataset as shown in Table 4. In addition, to demonstrating the efficiency of the weighted binary cross-entropy loss function over the conventional binary cross-entropy loss function, we additionally performed an experiment in the case of equal weight values, i.e., w_0_ of 0.50 and w_1_ of 0.50. The detailed experimental results are given in Table 6 for both the ResNet50-based network and the Inception-based network. As shown in Table 6, using the ResNet50-based network, we obtained an overall accuracy of about 87.778% with the sensitivity of 91.356% and specificity of 64.018% in the case of using the conventional binary cross-entropy loss function. These experimental results are little different from those reported by Nguyen et al. [36]. The reason is caused by the unstableness of the training process in which the network parameters were randomly initialized at the beginning of the training process at some new layers as shown in Table 2 and Table 3. Using the weighted binary cross-entropy loss function, we obtained an overall accuracy of 82.412% with a sensitivity of 83.950% and specificity of 72.524%. As we can see from these experimental results, the difference between the sensitivity and specificity in the case of using conventional binary cross-entropy loss function was about 27.338% (91.356%–64.018%). This result demonstrates that there was a bias in the classification result using the conventional binary cross-entropy loss function. Using the proposed weighted binary cross-entropy loss function, the difference between the sensitivity and specificity was much more reduced to about 11.426% (83.950%–72.524%). This result indicates that the bias was much more reduced by using the weighted cross-entropy loss function compared to the conventional binary cross-entropy loss function.

A similar phenomenon also occurred in our experiments with the Inception-based network. Using the conventional binary cross-entropy loss function, we obtained an overall classification accuracy of 81.506% with a sensitivity of 83.406% and a specificity of 68.760%. Using the proposed weighted binary cross-entropy loss function, we obtained an overall classification accuracy of about 80.792% with a sensitivity of 81.842% and a specificity of 74.016%. Similar to the experimental results by ResNet50-based network, the difference between the sensitivity and specificity was about 14.646% (83.460%–68.760%) in the case of using the conventional binary cross-entropy loss function that was much larger than the 7.826% (81.842%–74.016%) obtained in the case of using weighted binary cross-entropy loss function. Through these experimental results with the ResNet50-based network and Inception-based network, we could see that the proposed weighted binary cross-entropy loss function was more efficient for reducing the overfitting problem by reducing the difference between the sensitivity and specificity of the testing dataset.

Based on the trained models obtained by training the ResNet50-based network and Inception-based network, we further performed experiments by combining the classification results of these two models to investigate the enhancement ability of the combined network compared to the individual model. As explained in Section 2.4.2, we used three combination methods, including the MIN, MAX, and SUM rule, to combine the results of ResNet50-based network and Inception-based network as shown in Equations (2)–(4). The detailed experimental results are given in Table 7. Again, we performed experiments for the two cases of with and without the proposed weighted binary cross-entropy loss function. As shown in Table 7, we obtained the overall classification accuracy of 83.938%, 90.603%, and 82.677% for the case of using MIN, MAX, and SUM rule, respectively, with the use of the conventional binary cross-entropy loss function. The highest overall classification accuracy of 90.603% that was obtained using the MAX combination rule was much higher than the 87.778% obtained by using only ResNet50-based network or 81.506% using the Inception-based network. This result demonstrates that the combination of the results of the two networks helped to enhance the classification performance of our problem. In addition, the difference between the sensitivity and specificity using the MAX rule was about 36.728% (95.446%–58.718%). Similarly, we obtained an overall accuracy of 75.200%, 91.192%, and 78.709% for the case of using MIN, MAX, and SUM rule, respectively, with the use of the proposed weighted binary cross-entropy loss function. Again, the best classification accuracy was obtained using the MAX combination rule with the accuracy of about 91.192%. This classification accuracy was the highest accuracy among those obtained by only ResNet50-based model, Inception-based model even using conventional binary cross-entropy loss function or the proposed weighted binary cross-entropy loss function. In addition, the difference between the sensitivity and specificity was reduced to 29.396% (95.083%–65.687%), which was smaller than the 36.728% obtained using the conventional binary cross-entropy loss function. Through these experimental results, we could conclude that the combination of the multiple CNN networks could help to enhance the classification accuracy of the thyroid nodule classification, and the MAX rule outperformed the MIN and SUM rule for combining the results of individual models. In addition, the weighted binary cross-entropy loss function was efficient for reducing the overfitting problem caused by the unbalanced training samples of the target classes in the training data.

### 3.4. Classification Results by the Proposed Method

Based on our experimental results in Section 3.3, we finally performed the experiments to measure the performance of our proposed method as explained in Section 2.1 and Figure 2. The detailed experimental results are given in Table 8. Similar to the experiments in Section 3.3, we performed our experiments for two cases of with and without the proposed weighted binary cross-entropy loss function. For the case of the conventional binary cross-entropy loss function, we obtained overall classification accuracies of 86.928%, 90.603%, and 86.073% for the cases using MIN, MAX, and SUM rules, respectively. Compared with the classification results in Table 6 and Table 7, we see that the proposed method enhanced the classification results for the cases of MIN and SUM rules. For the case of MAX rule, the proposed method produced the same classification accuracy as the combination of multiple CNN models, which was still much higher than the performance of individual CNN models.

For the case of using the proposed weighted binary cross-entropy loss function, our proposed method produced classification accuracies of 83.517%, 92.051%, and 85.286%, for the MIN, MAX, and SUM rules, respectively. These classification accuracies were higher than those produced by individual CNN models and the combination of them as shown in Table 6 and Table 7. Especially, the highest classification accuracy of about 92.051% obtained by using the proposed method with a MAX combination rule was the highest classification result we obtained in all of our experiments in Table 6, Table 7 and Table 8. Compared to the case of using our proposed method but with the conventional binary cross-entropy loss function, the classification accuracy using our proposed method was also higher (92.051% versus 90.603%). This result again confirmed that our proposed method with the weighted binary cross-entropy loss function was efficient for reducing the overfitting problem, and consequently, enhancing the classification accuracy.

### 3.5. Performance Comparisons of Proposed Method with the State-of-the Art Methods

As explained in Section 1, there have been several previous studies that proposed their methods for solving the thyroid nodule classification problem. As one of the earliest studies, Zhu et al. [21] used the ResNet18-based network for the problem. To reduce the effect of overfitting, the transfer learning technique was applied, and they reported a classification accuracy of about 84.00% using the TDID dataset. To deal with the change in nodule sizes, Chi et al. [23] used the GoogLeNet, another name for the Inception network, for the problem. Using the method by Chi et al. [23], Nguyen et al. [36] evaluated the classification performance with the TDID dataset and reported an accuracy of about 79.36% in their experiments. In the study by Sundar et al. [28], they additionally performed experiments with the VGG16-based network for the thyroid nodule classification problem using their dataset. Using the VGG16-based network, a classification accuracy of 77.57% was obtained using the TDID dataset [36]. These mentioned studies have a similar characteristic in that they used a single CNN network with or without the transfer learning technique for the ultrasound image-based thyroid nodule classification problem. As a result, the performance of these studies depended extensively on the architecture of the selected CNN network as well as the training data. Most recently, Nguyen et al. [36] proposed a method based on a cascade classifier architecture that employs both handcrafted and deep learning-based methods. In that study, they first classified the input images using information extracted in the frequency domain. After that, the ambiguous samples were further processed by a deep learning-based network. The advantage of the study by Nguyen et al. [36] is that they combined information in both the frequency and spatial domains for the classification problem. However, they did not consider the difference in deep learning network architectures as well as the imbalance of image samples in the target classes as we did in this study. Nguyen et al. [36] reported a high classification accuracy of about 90.88% using their proposed method with the TDID dataset. Compared to the mentioned classification results by the previous studies, our proposed method produced much better classification accuracy. As shown in Section 3.4, our proposed method produced a classification accuracy of 92.051%. In Table 9, we summarized the previous classification performances in comparison with our proposed method. From the result in this table, we could conclude that our proposed method outperformed the previous studies for the ultrasound image-based thyroid nodule classification problem.

### 3.6. Analysis and Discussion

**Figure 9:**
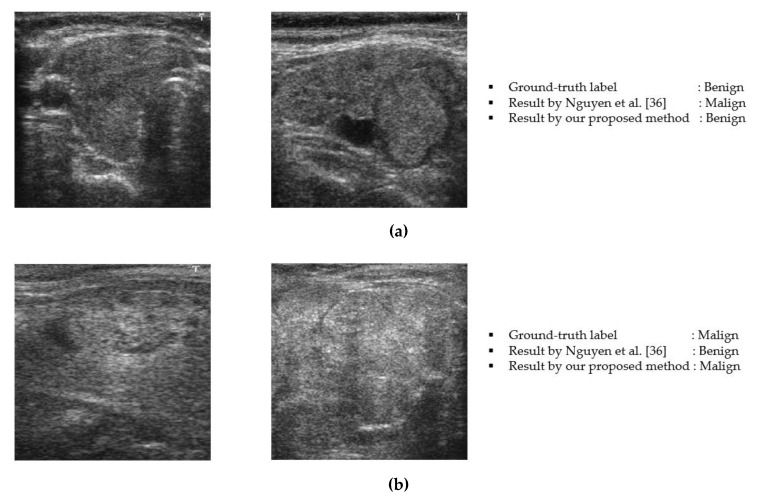
Example results obtained by our proposed method: (**a**) example results of the benign case and (**b**) example results of the malign case.

As shown in Table 9, our proposed method outperformed all of the methods presented in the previous studies using the TDID dataset. To get a deep visualization about the performance of our proposed method compared to a previous study by Nguyen et al. [36], we show some example classification results performed by both studies in Figure 9. In Figure 9a, we show the cases in which the ground-truth benign case images were incorrectly classified in the study by Nguyen et al. [36]. However, using our proposed method, we correctly classified them as benign cases. As we can observe from these images, although it is hard to label them as benign or malign case based on human perception as well as the system by Nguyen et al. [36], our proposed method can still recognize them as benign case images. Similar to Figure 9a but with examples of the malign case, Figure 9b shows the example classification results of malign case images. As shown in this figure, our proposed method also correctly classified them as the malign cases, while the method by Nguyen et al. [36] produced incorrect classification labels. By human perception, we could find that these images contain nodules with the calcification phenomenon (white blob region inside a round region (nodule)) that indicates that they should be malign case images. However, the method by Nguyen et al. [36] made an incorrect decision. This example shows that our proposed method was more effective than the method used in the study by Nguyen et al. [36]. Through this example and our experimental results in Section 3.5, we concluded that our proposed method was more effective than the previous studies for the thyroid nodule classification problem using ultrasound images.

As the final experiment in our study, we measured the processing time of our proposed method to evaluate the real system applicability of our algorithm. For this purpose, we used a desktop computer with an Intel Core i7-6700 CPU, working clock of 3.4 GHz with 64 GB of RAM memory. To speed up the deep learning networks, we used a GPU, namely GeForce Titan X, to run the inference of the two deep learning models [58]. To implement our algorithm, we used Python programming language with the Tensorflow library for implementing the deep CNN networks [59]. The consequent experimental results are given in Table 10. As shown in Section 2, our proposed method mainly consists of three main steps, which include the preprocessing step, a coarse classification by FFT-based method, and a fine classification by the combination of ResNet and InceptionNet. As shown in Table 10, it took about 11.4646 ms for the preprocessing step (thyroid region extraction and normalization), 5.093 ms for classifying the input image using FFT-based method, 17.525 ms for running the ResNet50-based network, and 23.178 ms for running the Inception-based network. As shown in Figure 2 and Section 2.1, our proposed method could produce the final decision in two scenarios. First, with the easy input samples, our proposed method only used the preprocessing and FFT-based steps to produce its decision. For difficult (complex) samples, our proposed method must invoke the fine classification steps based on deep learning networks. As a result, it takes at least 16.739 ms (11.646 + 5.093) and at most 57.442 ms (11.646 + 5.093 + 17.525 + 23.178) to produce a final prediction by our proposed method. In other words, our proposed method could operate at a speed ranging from 17.4 (1000 ÷57.442) to 59.7 (1000 ÷16.739) fps. Averagely, we could conclude that our proposed method could operate at a speed of about 38 fps. Through experimental result, we see that our proposed method was suitable for real-system application using a desktop computer.

## 4. Conclusions

In this study, we enhanced the classification performance of the ultrasound imaging-based thyroid nodule classification system by analyzing captured images of the thyroid region in two domains, i.e., the spatial domain using the deep learning-based method, and frequency domain using the fast Fourier-based method. Compared to the previous studies, we used two different CNN architectures, which were different in depth and network structures in this study to analyze an ultrasound thyroid image. As a result, the input ultrasound thyroid image was better analyzed compared to the single network as used in the previous studies. Finally, by combining the classification results of two CNN networks, we enhanced the overall performance of the classification system compared to the previous studies. In addition, we applied the weighted binary cross-entropy loss function for learning the CNN models instead of the conventional cross-entropy loss function to reduce the effects of the unbalanced training samples in the training procedure, and consequently reduce the effect of the under/overfitting problem. Through experiments with the public TDID dataset, we proved that our proposed method could give more accurate predictions/suggestions for doctors (radiologists) when diagnosing thyroid nodule problems than the previous studies.

## Figures and Tables

**Table 1:** Summary of the previous studies on the ultrasound thyroid nodule image classification problem.

Category	Method	Strength	Weakness
Handcrafted-based Methods	- Classification is implemented using extracted image features via human-designed methods [22,24,26,31]	- Easy to implement. - Does not require high-performance hardware devices	- Low classification accuracy
Deep learning-based methods	- Fine-tuning an existing CNN network for classification [21,23,28] - Extracts image features using a pretrained CNN network while classification is implemented using an SVM [28] - Combines detection and classification based on a CNN network [27]	- Utilizes the power of deep learning and transfer learning methods - Higher accuracy than handcrafted-based methods	- There is room for enhancing classification performance
Fusion of deep and handcrafted-based methods	- Extracts image features from both spatial and frequency domains for classification problem [36]	- Applies a cascade classifier scheme to enhance classification performance using handcrafted and deep features	- More complicated and takes longer processing time than using a single method (FFT-based or CNN-based methods)
	- Extracts image information from both spatial and frequency domains for classification problem - Combines classification results by multiple CNN models to enhance classification performance - Reduces the effect of unbalanced training samples of CNN network by using weighted cross-entropy loss function. (Proposed method)	- Analyzes the ultrasound thyroid images using different architectures of CNN network - Enhances the classification results compared to the use of single CNN architecture	- Requires strong hardware equipment to run multiple CNN networks - Takes longer processing time than the previous studies.

**Table 2:** ResNet50-based CNN architecture used in our experiments.

Layer	Input Shape	Output Shape	Number of Parameters
Convolution Layers by ResNet-50 Network	(224, 224, 3)	(7, 7, 2048)	23,587,712
Global Average Pooling	(7, 7, 2048)	2048	0
Batch Normalization	2048	2048	8192
Dropout	2048	2048	0
Output Layer (Dense layer)	2048	2	4098

**Table 3:** Inception-based CNN architecture used in our experiments.

Layer	Input Shape	Output Shape	Number of Parameters
Convolution Layers by Inception Network	(224, 224, 3)	(5, 5, 2048)	21,802,784
Global Average Pooling	(5, 5, 2048)	2048	0
Batch Normalization	2048	2048	8192
Dropout	2048	2048	0
Output Layer (Dense layer)	2048	2	4098

**Table 4:** Description of the TDID dataset used in our experiments (each number means the number of patients).

Benign Case		Malign Case		Total
Training Data	Testing Data	Training Data	Testing Data	
41	11	196	50	298

**Table 5:** Parameters for training CNN models in our study.

Optimizer	Number of Epochs	Batch Size	Initial Learning Rate	Stop Criteria
Adam	30	32	0.0001	End of Epochs

**Table 6:** Classification performance of the individual CNN network using the TDID dataset (unit: %).

Method	ResNet50-Based Network			Inception-Based Network		
	Accuracy	Sensitivity	Specificity	Accuracy	Sensitivity	Specificity
Using BCE [39,40,41,42]	87.778	91.356	64.018	81.506	83.406	68.760
Using wBCE (proposed method)	82.412	83.950	72.524	80.792	81.842	74.016

**Table 7:** Classification performance by combining the two CNN networks using MIN, MAX, and SUM rules (unit: %).

Method	MIN Rule			MAX Rule			SUM Rule		
	Accuracy	Sensitivity	Specificity	Accuracy	Sensitivity	Specificity	Accuracy	Sensitivity	Specificity
Using BCE [39,40,41,42]	83.938	85.868	71.142	90.603	95.446	58.718	82.677	83.894	74.219
Using wBCE (proposed method)	75.200	74.859	77.226	91.192	95.083	65.687	78.709	79.167	75.967

**Table 8:** Classification performance of our proposed method using the TDID dataset with MIN, MAX, and SUM rules (unit: %).

Method	MIN Rule			MAX Rule			SUM Rule		
	Accuracy	Sensitivity	Specificity	Accuracy	Sensitivity	Specificity	Accuracy	Sensitivity	Specificity
Using BCE [39,40,41,42]	86.928	89.331	71.142	90.603	95.446	58.718	86.073	87.831	74.219
Using wBCE (proposed method)	83.517	84.466	77.226	92.051	96.072	65.687	85.286	86.748	75.967

**Table 9:** Comparison of the overall accuracy of the previous studies and our proposed method with the TDID dataset (unit: %).

Methods		Accuracy
Zhu et al. [21]		84.00
Chi et al. [23]		79.36
Sundar et al. [28]	VGG16	77.57
	GoogLeNet	79.36
Nguyen et al. [36]		90.88
Proposed Method		92.05

**Table 10:** Processing time of our proposed method (unit: ms).

Preprocessing Step.	FFT-Based Classification	ResNet50-Based Classification	Inception-Based Classification	Total
11.646	5.093	17.525	23.178	57.442
